# Effects of dietary supplementation with different levels of rose pomace on rumen bacterial diversity and fermentation, and longissimus dorsi fatty acids in Hu sheep

**DOI:** 10.3389/fmicb.2025.1604624

**Published:** 2025-07-23

**Authors:** Linjiao He, Ruirui Tian, Ziting Wang, Jinlong Li, Shan Zhang, Zhijun Zhang

**Affiliations:** ^1^Feed Research Institute of Xinjiang Uygur Autonomous Region Academy of Animal Sciences, Urumgi, China; ^2^College of Animal Science, Xinjiang Agricultural University, Urumqi, China; ^3^Key Laboratory of Herbivorous Livestock Feed Biotechnology, Urumgi, China; ^4^College of Grassland Science, Xinjiang Agricultural University, Urumqi, China

**Keywords:** rose pomace, growth performance, rumen, fatty acids, bacterial diversity, bacteroidota

## Abstract

To address the underutilization of rose processing byproducts and meet the demand for antibiotic-free meat production, this study examined the effects of dietary supplementation with rose pomace (RP) on growth performance, rumen fermentation, bacterial diversity, and longissimus dorsi amino acid and fatty acid profiles in Hu sheep. Forty male Hu sheep were randomly assigned to four groups (*n* = 10): a control group (RP0) with a basal diet and three treatment groups supplemented with 2, 4, or 8% RP (RP2, RP4, RP8). Results showed that RP8 had significantly higher dry matter intake (DMI) than RP2 (*P* ≤ 0.05). Rumen fermentation analysis indicated higher acetate in RP4 than RP0 (*P* ≤ 0.05), while propionate and total volatile fatty acids (TVFA) were lower in RP0 than in all RP groups (*P* ≤ 0.05). RP8 exhibited significantly elevated isobutyrate (*P* ≤ 0.05) and isovalerate (*P* ≤ 0.01), with valerate levels higher in RP4 and RP8 than RP0 (*P* ≤ 0.05). Microbial analysis revealed increased Bacteroidota and reduced Patescibacteria in RP8 (*P* ≤ 0.05). RP8 also showed higher *Rikenellaceae_RC9_gut_group* abundance (*P* ≤ 0.05). In longissimus dorsi muscle, RP4 had significantly higher C18:3N3, N-3 PUFAs, and C20:2N6 than RP0 and RP8 (*P* ≤ 0.05). These findings suggest RP modulates rumen microbiota and fermentation, enhancing beneficial fatty acid deposition in lamb meat. An RP supplementation level of 2–4% yielded optimal results, providing valuable insights for sheep farmers seeking functional feed additives.

## 1 Introduction

Lamb meat is a high-quality protein source that is also low in fat and cholesterol (Ding et al., 2024). With the improvement in both scale and technological sophistication of China’s intensive livestock farming, the production performance of meat sheep has been continuously enhanced. However, intensive farming practices are often accompanied by increased stress on animals and reduced meat quality due to factors such as management approaches, stocking density, and disease control measures (Wang et al., 2021). Under the current policy of a comprehensive ban on antibiotics as feed additives in China’s livestock industry, the development of novel plant-derived natural additives holds significant importance for promoting healthy lamb production and enhancing the quality of animal-derived products.

The rugosa rose (*Rosa rugosa* Thunb.) is an herbaceous perennial belonging to the Rosaceae family, primarily cultivated for its flower buds which serve as the main raw material for the extraction of essential oil of roses. Rose flowers have been regarded as medicinal plants for millennia with biological functions including the regulation of oxidative stress and lipid metabolism (Hegde et al., 2022). The predominant industrial application of rugosa roses is for essential oil production, and, with a yield ranging from 0.03 to 0.07% (w/w), the extraction process generates a large amount of rose pomace (RP) as a byproduct (Allahyari et al., 2021; Katekar et al., 2022). Historically, except for a minor portion utilized as natural air-dried fuel, the majority of RP has been indiscriminately discarded. This practice not only represents a significant waste of a bioresource, but can also cause environmental pollution due to mold proliferation and the malodorous emissions produced during spontaneous fermentation (Slavov et al., 2017).

While roses currently demonstrate diverse applications, the effective management of processing byproducts remains a critical challenge, as with most agricultural products. Rose pomace—the residual material following essential oil extraction—is particularly valuable for its rich dietary fiber content and abundance of bioactive compounds including polyphenols, flavonoids, and polysaccharides (Milala et al., 2023), with obvious potential for enhancing animal health. Recent years have witnessed growing research interest in rose-derived products, coinciding with the global shift toward demand for higher quality in food and the adoption of green practices in raising food animals. Loetscher et al. (2013) demonstrated that dietary supplementation with rosehips in broilers significantly enhanced daily dry matter intake (DMI) and average daily gain (ADG). Konca et al. (2021) reported that 5–10% (w/w) rosehip inclusion in Japanese quail (*Coturnix japonica*) diets increased egg weight, improved albumen quality, and enhanced yolk pigmentation. Despite these findings, current research on rose-derived feed additives remains constrained to post-anthesis fruit byproducts in poultry systems, with particular emphasis on laying performance and egg quality parameters (Vlaicu et al., 2022). Notably, no peer-reviewed studies have investigated the effects of the rose flower byproduct, pomace, on ruminant production. This study therefore pioneers the systematic evaluation of *Rosa rugosa* pomace as a novel plant-derived additive in lamb production systems, with tripartite investigation of (a) growth performance parameters (DMI, ADG, feed conversion ratio), (b) rumen microbiome modulation via 16S rRNA sequencing, and (c) meat quality biomarkers including fatty acid profiles (GC-MS quantified) and amino acid composition (HPLC analyzed). The resultant data provide critical insights into sustainable valorization of rose processing waste, simultaneously addressing the dual challenges of agro-industrial byproduct utilization and premium meat production in antibiotic-free farming systems.

## 2 Materials and methods

### 2.1 Ethics Statement

All animal care and experimental protocols were conducted in strict compliance with the Guidelines for the Care and Use of Laboratory Animals issued by the Ministry of Science and Technology of China and ethically approved by the Institutional Animal Care and Use Committee (IACUC) of the Xinjiang Uygur Autonomous Region Academy of Animal Science (Approval No. 320240107; Urumgi, China).

### 2.2 Experimental materials

**FIGURE 1:**
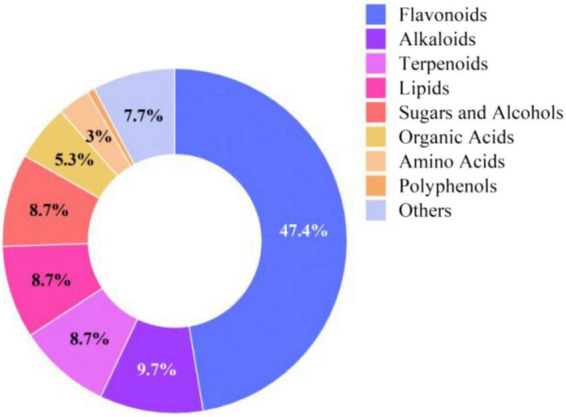
Relative abundance of bioactive compounds in rose pomace.

The *Rosa rugosa* Thunb. pomace (residue from essential oil extraction) was provided by Xinjiang Yutian Guimi Biotechnology Co., Ltd. (Yutian County, Hotan Prefecture, China; GPS coordinates: 36°46′N, 81°50′E). The proximate composition (dry matter basis) of the RP is detailed in Table 1. The active components in rose pomace were analyzed using ultra-performance liquid chromatography coupled with tandem mass spectrometry (UPLC-ESI-MS/MS). After sample pretreatment through solvent extraction and filtration, chromatographic separation was achieved with a reverse-phase C18 column employing gradient elution of acidified aqueous and organic phases. Mass spectrometric detection was performed in both positive and negative ionization modes with multiple reaction monitoring, simultaneous qualification and quantification of diverse bioactive compounds including flavonoids, phenolic acids and terpenoids. As illustrated in Figure 1, flavonoids constituted the predominant bioactive components (relative abundance: 47.4%), followed by terpenoids (9.7%), based on quantification of normalized peak areas.

**TABLE 1:** Proximate composition of rose pomace (% dry matter basis).

Item	Dry matter (DM)	Crude protein (CP)	Crude fat (EE)	Neutral detergent fiber (NDF)	Acid detergent fiber (ADF)	Calcium (Ca)	Phosphorus (P)
Rose pomace	96.21	3.59	1.87	64.86	44.69	2.19	0.14

Data presented as mean values from triplicate assays following the AOAC official method, 934.01.

### 2.3 Experimental timeline and location

The trial was conducted from January to March 2024 at the Hu Sheep Feedlot operated by Xinjiang Taihe Agricultural and Animal Husbandry Technology Co., Ltd. (Kashgar, China; 39°46′N, 78°36′E), a facility certified under the GB/T 20014–2018 good agricultural practice standards.

### 2.4 Experimental design

Forty healthy male purebred Hu sheep (*Ovis aries*), 3–4 months of age, with comparable initial body weight (27.42 ± 1.80 kg; mean ± SD), were selected and randomly allocated into four groups (*n* = 10/group) after a 14-day quarantine period with ivermectin anthelminthic treatment (0.2 mg/kg BW). The experimental sheep were divided into four enclosures, with 10 sheep in each enclosure, and they were raised separately in their corresponding enclosures. The dietary treatments included:

RP0 (Control): Basal pelleted diet
RP2: Basal diet + 2% RP (DM basis)
RP4: Basal diet + 4% RP (DM basis)
RP8: Basal diet + 8% RP (DM basis)

The corresponding rose pomace in each group was crushed and mixed with other feed ingredients, then extruded into cylindrical pellets with a standardized 8mm diameter using a granulation device (model SZLH508; Jiangsu Liangyou Zhengda Co., Ltd., Suzhou, China) to facilitate lamb consumption.

The 67-day trial comprised a 7-day adaptation period, a gradual transition from the maintenance diet to the experimental diets, and a 60-day feeding trial, with ad libitum feeding of diets formulated according to NRC (2007) nutrient requirements for growing lambs. The composition of the basal diet is given in Table 2 and the analyzed nutritional profiles of the experimental diets are presented in Table 3. During the trial period, fresh water and mineral blocks were provided at all times. Mineral blocks were also replenished monthly for every lamb group.

**TABLE 2:** Composition of basal diet (% dry matter basis).

Item	RP0	RP2	RP4	RP8
Corn stalks	17.25	17.25	17.25	17.25
Cotton stalks	16.89	16.89	16.89	16.89
Cotton leaves	6.35	6.35	6.35	6.35
Corn grain	30.00	30.00	30.00	30.00
Wheat bran	8.23	8.23	8.23	8.23
Cottonseed meal	16.86	16.86	16.86	16.86
Ammonium chloride (NH_4_Cl)	0.10	0.10	0.10	0.10
Sodium chloride (NaCl)	0.82	0.82	0.82	0.82
Sodium bicarbonate (NaHCO_3_)	1.37	1.37	1.37	1.37
Premix^1^	2.13	2.13	2.13	2.13
Total	100	100	100	100
Rose pomace	0.00	2.00	4.00	8.00

^1^The premix provided (per kg of diet): Vit A 150,000 IU; Vit D*3* 56,500 IU, Vit E 8,000 IU; selenium (as sodium selenite) 14 mg, iodine (as potassium iodide) 80 mg, copper (as copper sulfate) 290 mg; manganese (as manganese sulfate) 1,925 mg; zinc (as zinc oxide) 2,050 mg, cobalt (as cobalt sulfate) 24 mg.

**TABLE 3:** Analyzed nutrient composition of experimental diets (% dry matter basis).

Item	RP0	RP2	RP4	RP8
ME (MJ/kg)^1^	10.09	10.03	9.95	9.80
Crude protein	13.97	13.76	13.55	13.12
Neutral detergent fiber	36.74	37.28	37.77	38.61
Acidic detergent fiber	23.07	23.48	23.87	24.53
Crude fat	3.26	3.22	3.2	3.14
Calcium	0.55	0.58	0.61	0.67
Phosphorus	0.39	0.38	0.38	0.37

^1^ME, metabolizable energy; values calculated using NRC (2007) equations. The crude protein content (Method 920.37), neutral detergent fiber (Method 973.18), acid detergent fiber (Method 973.18), ash (Method 942.05), calcium (Method 927.02), and phosphorus (Method 965.17) in the feed were determined by the AOAC method (Latimer, 2023).

### 2.5 Feeding management

Prior to trial commencement, all pens were thoroughly cleaned and disinfected using 2% sodium hypochlorite solution. Throughout the experimental period, lambs were group-housed in identical environmental conditions with ad libitum access to feed (offered at 09:00 and 18:00 daily) and fresh water. To ensure consistent nutrient intake, daily feed residues were maintained at 15% of the offered quantity through gravimetric adjustment.

### 2.6 Growth performance and slaughter protocol

Body weights were recorded before morning feeding on day 1 (initial) and day 61 (final) of the formal trial period. Average daily gain (ADG) was calculated as:


(1) $$\mathrm{ADG}\,\left(g/\mathrm{day}\right)=\frac{\mathrm{Final}\,\mathrm{BW}\,\left(\mathrm{kg}\right)-\mathrm{Initial}\,\mathrm{BW}\,\left(\mathrm{kg}\right)}{60}\times 1000$$

Following the Guidelines for the Ethical Treatment of Experimental Animals, lambs were humanely slaughtered through a standardized procedure. After a 12-h fasting period with free access to water, pre-slaughter live weight was measured. Exsanguination was performed via simultaneous severance of the carotid artery and jugular vein, after which the head, hooves, skin, and internal organs (excluding perirenal fat) were removed. The carcass weight was immediately recorded, and the dressing percentage was calculated as:


(2) $$\mathrm{Slaughter}\mathrm{rate}\left(\%\right)=\frac{\mathrm{Carcass}\,\mathrm{weight}\,\left(\mathrm{kg}\right)}{\mathrm{Liveweight}\,\mathrm{before}\,\mathrm{slaughter}\,\left(\mathrm{kg}\right)}\times 100\%$$

### 2.7 Rumen fluid sampling and analysis

Rumen content samples were collected via oral intubation by inserting a rumen fluid collection tube through the mouth into the rumen, followed by syringe aspiration and immediate filtration through four layers of sterile gauze to separate particulate matter. To minimize cross-contamination from oral microbiota, the initial 50 mL of filtrate was discarded. Subsequently, 10 mL of clarified rumen fluid was aliquoted into sterile cryovials and snap-frozen in liquid nitrogen for preservation. Prior to analysis, thawed samples were centrifuged at 15,000 × g for 10 min at 4 °C. The resultant supernatants were analyzed for ammonia nitrogen (NH_3_-N) using the phenol-hypochlorite method (Broderick and Kang, 1980) and the absorbance at 630 nm was measured with a UV-1800 spectrophotometer (Shimadzu Corp., Kyoto, Japan). Volatile fatty acid (VFA) concentrations were determined by gas chromatography (Clarke, 1993) (GC-450, Varian Inc., United States), with detection limits of 0.01 mm for acetate, propionate, butyrate, isobutyrate, valerate, and isovalerate.

### 2.8 Determination of amino acids in meat

The determination of amino acids refers to Li J. et al. (2025). Freeze-dried tissue samples (100 mg) were homogenized in 2.0 mL microcentrifuge tubes with 1.2 mL of 10% (w/v) sulfosalicylic acid solution. Following vortex mixing for 3 min, the homogenate was centrifuged at 13,500 × g for 15 min at 4°C. The supernatant was filtered through 0.22 μm polyethersulfone (PES) membranes and transferred to 2.0 mL glass threaded vials with PTFE/silicone septa. Fatty acid concentrations were quantified using UPLC-ESI-MS/MS (Waters Acquity I-Class PLUS; Applied Biosystems QTRAP 6500+) with an HSS-T3 column (1.8 μm, 2.1 × 100 mm). The mobile phase consisted of (A) 0.1% formic acid/5 mM ammonium acetate in water and (B) 0.1% formic acid in acetonitrile, employing a 14-min gradient (2–98% B; 0.35 mL/min, 50°C). ESI parameters were maintained at 550°C and ± 5,500/4,500 V with curtain gas (35 psi). Analytes were detected via optimized MRM transitions, and concentrations were determined by normalizing peak areas to total area using external calibration curves.

### 2.9 Determination of fatty acids in meat

The determination of amino acids refers to Li J. et al. (2025). Total fatty acid extraction from frozen meat samples was performed using the direct methylation protocol described by O’Fallon et al. (2007), involving sequential saponification (2 N KOH/methanol, 55°C, 1.5 h) and methylation (14% boron trifluoride/methanol, 100°C, 30 min). Fatty acid methyl esters (FAMEs) were analyzed using a gas chromatograph (GC-450, Varian Inc., United States). Peak identification was based on retention time alignment with certified FAME standards (C4-C24 methyl esters, CRM47885, Sigma-Aldrich, US). Individual fatty acid concentrations were quantified using external calibration curves generated from five-point serial dilutions of the standard mixture.

### 2.10 DNA extraction, PCR amplification, and rumen bacterial sequencing analysis

Bacterial DNA was extracted from rumen fluid using the TGuide S96 Magnetic Soil/Stool DNA kit (Tiangen Biotech, Beijing, China) according to the manufacturer’s instructions. Full-length 16S rRNA gene amplification was performed with the primers 27F (5′-AGRGTTTGATYNTGGCTCAG-3′) and 149 2R (5′-TASGGHTACCTTGTTASGACTT-3′). PCR conditions included an initial denaturation at 95°C for 3 min, followed by 30 cycles of denaturation (95°C, 30 s), annealing (55°C, 30 s), and extension (72°C, 90 s), with a final extension at 72°C for 7 min.

For DNA library preparation, total DNA was amplified using barcoded 16S-specific primers (27F/1492R). PCR products were purified, quantified, normalized, and ligated into SMRT Bell libraries. After passing quality control, libraries were sequenced on the PacBio Sequel II platform. Raw sequencing data (.bam format) were processed through the smrtlink pipeline to generate circular consensus sequencing (CCS) reads. CCS sequences were demultiplexed using lima v1.7.0 to obtain raw-CCS data, followed by primer trimming and length filtering with cutadapt v1.9.1 to generate clean-CCS sequences. Chimeric sequences were removed using UCHIME v4.2, yielding effective-CCS sequences. All downstream analyses were conducted on the Biomarker Biotechnology Cloud Platform.^1^

### 2.11 Statistical analysis

Experimental data were initially processed using Microsoft Excel 2013. Statistical analyses were performed with SPSS 26.0 software. One-way ANOVA followed by Duncan’s multiple range test was applied for comparisons among groups. Results are expressed as mean ± SEM. Statistical significance was defined as *P* ≤ 0.05, while *P* ≤ 0.01 was considered highly significant. A *P*-value between 0.05 and 0.10 was interpreted as indicative of a trend. Correlation analyses were visualized using Origin 2021 software.

## 3 Results and discussion

### 3.1 Growth performance and slaughter traits

As shown in Tables 4, 5, the dry matter intake (DMI) of the RP8 group was significantly higher than that of the RP2 group (*P* ≤ 0.05). No statistically significant differences (*P* > 0.05) were observed among groups for other growth performance or slaughter parameters.

**TABLE 4:** Effects of dietary rose pomace supplementation on growth performance of lambs (*n* = 10).

Item	RP0	RP2	RP4	RP8	SEM	*P*-value		
						Contrast	Linear	Quadratic
Initial weight, kg	27.40	27.65	27.03	27.47	0.273	0.886	0.897	0.997
Final weight, kg	43.31	43.12	42.72	43.23	0.503	0.979	0.864	0.799
ADG, kg/d	0.26	0.26	0.26	0.26	0.006	0.994	0.978	0.832
DMI, kg/d	1.76^ab^	1.69^b^	1.76^ab^	1.80^a^	0.014	0.050	0.106	0.063
F/G	6.68	6.52	6.70	6.85	0.055	0.214	0.159	0.162

^a,b^Different superscripts indicate significant differences within a row (*P* < 0.05). SEM is the pooled standard error between five groups; the *P*-value indicates significance. ADG: average daily gain; DMI: dry matter intake, F/G: feed-to-gain ratio.

**TABLE 5:** Effects of dietary rose pomace supplementation on slaughter performance of lambs (*n* = 5).

Item	RP0	RP2	RP4	RP8	SEM	*P*-value		
						Contrast	Linear	Quadratic
Carcass weight, kg	23.06	22.58	22.16	22.42	0.265	0.71	0.322	0.673
Slaughter rate, %	0.49	0.49	0.51	0.50	0.004	0.376	0.625	0.721

^a,b^Different superscripts indicate significant differences within a row (*P* < 0.05). SEM is the pooled standard error between five groups; the *P*-value indicates significance.

### 3.2 Rumen fermentation parameters

As presented in Table 6, increasing dietary rose pomace inclusion elicited linear responses in ruminal concentrations of acetate, propionate, isobutyrate, butyrate, isovalerate, valerate, and TVFA (*P* = 0.024, *P* = 0.004, *P* = 0.006, *P* = 0.05, *P* = 0.000, *P* = 0.003, *P* = 0.009), while NH_3_-N exhibited a quadratic change (*P* = 0.042). Notably, the RP4 group demonstrated significantly higher acetate levels compared to RP0 (*P* ≤ 0.05), whereas propionate and TVFA concentrations in RP0 were significantly lower than those in RP2, RP4, and RP8 (*P* ≤ 0.05). The RP8 group showed marked increases in isobutyrate (*P* ≤ 0.05) and isovalerate (*P* ≤ 0.01) relative to RP0, together with elevated valerate levels in both RP4 and RP8 groups (*P* ≤ 0.05).

**TABLE 6:** Effects of dietary rose pomace supplementation on rumen fermentation parameters in lambs (*n* = 6).

Item	RP0	RP2	RP4	RP8	SEM	*P*-value		
						Contrast	Linear	Quadratic
Acetate, mmol/L	19.69^b^	26.64^ab^	29.52^a^	25.95^ab^	1.305	0.043	0.024	0.088
Propionate, mmol/L	5.00^b^	6.94^a^	7.88^a^	7.23^a^	0.349	0.012	0.004	0.122
Isobutyrate, mmol/L	0.78^b^	0.90^ab^	0.96^ab^	1.14^a^	0.046	0.036	0.006	0.439
Butyrate, mmol/L	3.03	3.53	4.47	3.98	0.228	0.133	0.05	0.563
Isovalerate, mmol/L	1.18^cB^	1.58^bcAB^	1.74^bAB^	2.25^aA^	0.111	0.002	0.00	0.336
Valerate, mmol/L	0.44^b^	0.53^ab^	0.58^a^	0.64^a^	0.025	0.027	0.003	0.810
TVFA, mmol/L	30.11^b^	40.12^a^	45.15^a^	41.18^a^	1.913	0.026	0.009	0.137
A/P	3.91	3.91	3.72	3.61	0.071	0.365	0.127	0.435
NH_3_-N, mg/dL	23.00	16.77	19.17	23.8	1.311	0.191	0.670	0.042

^A,B^Different superscripts indicate significant differences within a row (*P* < 0.01). ^a,b,c^Different superscripts indicate significant differences within a row (*P* < 0.05). SEM is the pooled standard error between five groups; the *P*-value indicates significance. TVFA, total volatile fatty acids; A/P, acetate/propionate.

### 3.3 Rumen microbiota

**FIGURE 2:**
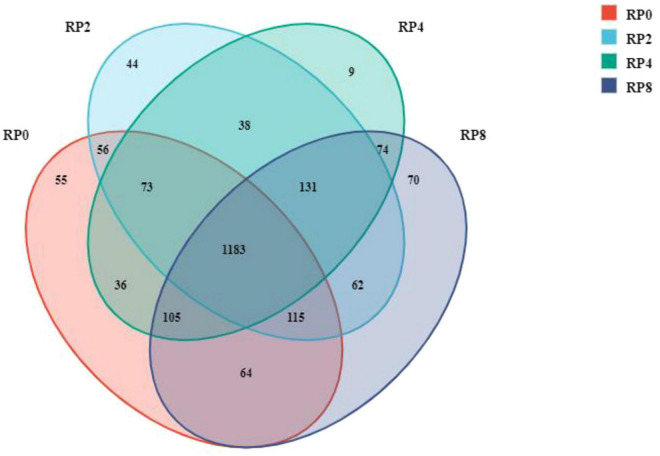
Venn diagram of OTU distribution among experimental groups (*n* = 6).

As shown in Figure 2, sequences were clustered at a 97% similarity threshold to define operational taxonomic units (OTUs). A total of 2,115 OTUs were identified across all four groups. The OTU counts for RP0, RP2, RP4, and RP8 groups were 1702, 1649, 1684, and 1687, respectively. Among these, 1183 OTUs (55.93% of total) were shared among all groups. Unique OTUs per group numbered 44 (2.08%), 9 (0.43%), 70 (3.31%), and 55 (2.60%) for RP0, RP2, RP4, and RP8, respectively, indicating relatively minor compositional differences in OTU distribution across dietary treatments.

Alpha-diversity analysis revealed no significant differences (*P* > 0.05) among groups for the ACE, Chao1, Simpson, and Shannon indices, or coverage (Table 7).

**TABLE 7:** Effects of dietary rose pomace supplementation on rumen microbial alpha-diversity in Hu sheep (*n* = 6).

Item	RP0	RP2	RP4	RP8	SEM	*P*-value		
						Contrast	Linear	Quadratic
ACE	1041.72	1143.03	1167.60	1224.08	38.419	0.421	0.104	0.969
Chao1	1035.34	1137.11	1106.21	1219.20	35.360	0.339	0.106	0.829
Simpson	0.95	0.98	0.94	0.96	0.010	0.432	0.921	0.684
Shannon	7.07	7.58	6.73	7.35	0.168	0.319	0.907	0.850
Coverage	0.98	0.97	0.97	0.97	0.001	0.428	0.168	0.575

SEM is the pooled standard error between five groups; the *P*-value indicates significance.

**FIGURE 3:**
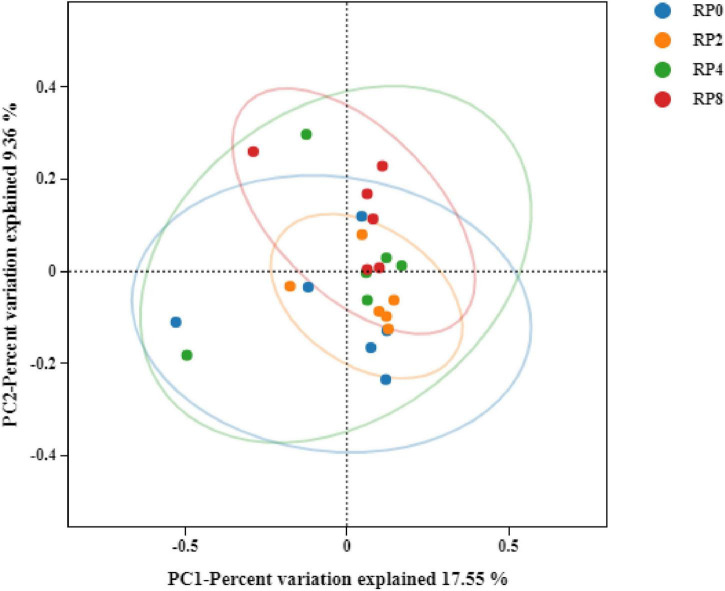
Principal coordinates analysis (PCoA) of rumen bacterial communities (*n* = 6).

Principal coordinates analysis (PCoA) based on Bray-Curtis dissimilarity at the OTU level demonstrated minimal separation among groups, with principal coordinate 1 (PC1) and PC2 explaining 17.55 and 9.36% of total variance, respectively (Figure 3). The close clustering of sample points across dietary treatments indicates that rose pomace supplementation exerted limited structural impacts on the rumen microbial ecosystem in Hu sheep.

As detailed in Table 8, linear responses were observed in the relative abundances of Firmicutes, Bacteroidota, and Patescibacteria with increasing RP inclusion (*P* = 0.016, *P* = 0.007, *P* = 0.014). Notably, the RP8 group exhibited an extremely significant elevation in Bacteroidota abundance compared to RP0 and RP2 (*P* ≤ 0.01), while showing reduced Patescibacteria levels relative to these groups (*P* ≤ 0.05). Marginal linear decreases (0.05 ≤ *P* ≤ 0.10) were detected for Planctomycetota (*P* = 0.076) and Desulfobacterota (*P* = 0.086). These shifts suggest selective modulation of rumen microbial ecology by dietary polyphenol-fiber interactions.

**TABLE 8:** Effects of dietary rose pomace supplementation on rumen bacterial composition at the phylum-level in Hu sheep (*n* = 6).

Item	RP0	RP2	RP4	RP8	SEM	*P*-value		
						Contrast	Linear	Quadratic
Firmicutes	61.93	59.18	56.31	46.08	2.256	0.058	0.016	0.199
Bacteroidota	28.89^b^	33.16^b^	37.63^ab^	46.47^a^	2.324	0.036	0.007	0.295
Verrucomicrobiota	1.48	2.73	1.24	2.19	0.261	0.164	0.613	0.523
Proteobacteria	1.14	2.17	1.55	2.28	0.287	0.475	0.244	0.793
Planctomycetota	3.95	0.64	0.89	0.61	0.685	0.245	0.076	0.364
Actinobacteriota	0.53	0.44	0.74	0.38	0.073	0.315	0.845	0.527
Desulfobacterota	0.60	0.46	0.27	0.40	0.055	0.203	0.086	0.465
Spirochaetota	0.33	0.16	0.32	0.49	0.075	0.529	0.498	0.205
Patescibacteria	0.41^a^	0.40^a^	0.21^ab^	0.11^b^	0.047	0.047	0.014	0.235
Fusobacteriota	0.05	0.08	0.40	0.37	0.089	0.367	0.138	0.791
Others	0.70	0.59	0.44	0.62	0.090	0.808	0.608	0.562

^a,b^Different superscripts indicate significant differences within a row (*P* < 0.05). SEM is the pooled standard error between five groups; the *P*-value indicates significance.

As delineated in Table 9, increasing dietary RP inclusion elicited a linear response (*P* = 0.030) in the relative abundance of the *Christensenellaceae_R-7_group*, while *Prevotella* exhibited a marginal linear trend (*P* = 0.076). Notably, *Rikenellaceae_RC9_gut_group* demonstrated a quadratic response (*P* = 0.001), characterized by an initial decrease followed by rebound, with the RP8 group showing extremely significant elevation compared to RP2 and RP4 (*P* ≤ 0.01), and significant increase over RP0 (*P* ≤ 0.05).

**TABLE 9:** Effects of dietary rose pomace supplementation on rumen bacterial composition at the genus-level in Hu sheep (*n* = 6).

Item	RP0	RP2	RP4	RP8	SEM	*P*-value		
						Contrast	Linear	Quadratic
*Prevotella*	13.4	18.5	24.47	22.99	2.201	0.289	0.076	0.741
*Christensenellaceae_R_7_group*	12.44	10.02	9.63	7.2	0.815	0.156	0.030	0.755
*Rikenellaceae_RC9_gut_group*	9.69^bAB^	5.58^bB^	6.69^bB^	16.36^aA^	1.267	0.004	0.087	0.001
*Lachnospiraceae_NK3A20_group*	10.42	4.31	10.64	4.5	2.247	0.631	0.534	0.91
*Uncultured_rumen_bacterium*	5.23	6.83	4.97	6.29	0.368	0.242	0.561	0.628
*Ruminococcus*	4.51	4.86	5.85	2.85	0.493	0.189	0.483	0.107
*Succiniclasticum*	3.41	3.28	2.24	2.96	0.345	0.659	0.447	0.774
*Selenomonas*	2.36	4.99	3.26	1.95	0.454	0.075	0.778	0.014
*Unclassified_Selenomonadaceae*	2.7	4.06	3.42	1.7	0.678	0.671	0.711	0.246
*NK4A214_group*	1.66	1.99	1.47	1.89	0.102	0.283	0.769	0.922
Others	34.17	35.59	27.35	31.29	1.82	0.414	0.341	0.926

^A,B^Different superscripts indicate significant differences within a row (*P* < 0.01). ^a,b^Different superscripts indicate significant differences within a row (*P* < 0.05). SEM is the pooled standard error between five groups; the *P*-value indicates significance.

**FIGURE 4:**
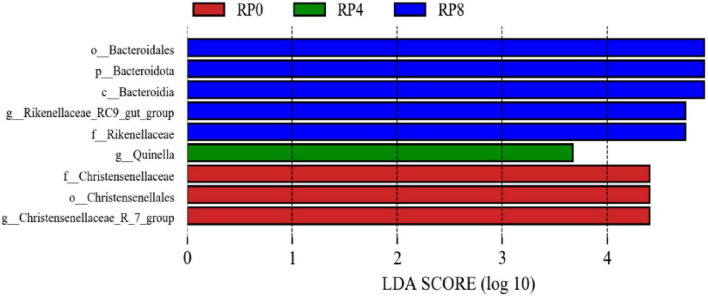
Taxonomic biomarkers identified by LEfSe analysis (LDA score ≥ 3.5).

As illustrated in Figure 4, linear discriminant analysis (LDA) with a threshold score ≥ 3.5 revealed differential microbial signatures across groups at the phylum-to-genus level. The control group (RP0) exhibited three taxa significantly influencing community structure, while RP2 showed no discriminative taxa. The RP4 and RP8 groups demonstrated one and five discriminative taxa, respectively, with the latter displaying the highest number of lineage-specific biomarkers, indicative of dose-dependent modulation of rumen microbiota by RP supplementation.

### 3.4 Meat quality attributes

As shown in Table 10, no significant differences (*P* > 0.05) were detected in the amino acid composition of longissimus dorsi muscle among the groups. Notably, histidine content exhibited a quadratic trend (*P* = 0.091) with increasing RP inclusion, suggesting a potential threshold effect of polyphenol-fiber interactions on this essential amino acid.

**TABLE 10:** Effects of dietary rose pomace supplementation on amino acid content in lamb longissimus dorsi muscle (mg/g) (*n* = 5).

Item	RP0	RP2	RP4	RP8	SEM	*P*-value		
						Contrast	Linear	Quadratic
**Essential amino acid**								
Methionine	47.26	57.77	50.61	56.13	3.267	0.679	0.455	0.680
Valine	54.58	66.81	66.59	63.21	2.566	0.308	0.175	0.188
Lysine	41.46	46.65	50.58	40.71	2.073	0.303	0.728	0.116
Isoleucine	41.86	51.36	46.89	48.35	2.144	0.494	0.335	0.361
Phenylalanine	122.14	152.38	128.81	139.12	8.734	0.670	0.620	0.516
Leucine	146.60	174.92	168.34	185.45	9.233	0.533	0.185	0.861
Tryptophan	6.71	8.45	7.60	7.65	0.369	0.458	0.407	0.255
Threonine	381.96	534.92	481.96	513.89	34.378	0.433	0.208	0.425
**Non-essential amino acid**								
Alanine	570.39	603.07	601.72	644.48	29.860	0.876	0.453	0.870
Arginine	239.84	243.19	240.52	220.38	13.266	0.938	0.689	0.646
Asparagine	46.02	55.01	50.16	49.68	2.152	0.564	0.585	0.267
Aspartate	137.50	123.60	241.19	147.18	28.435	0.480	0.572	0.745
Cysteine	9.61	8.66	11.36	11.27	0.833	0.635	0.397	0.577
Glutamine	932.15	725.65	1161.49	842.51	96.300	0.461	0.909	0.965
Glutamate	59.00	46.87	76.89	66.92	6.697	0.472	0.460	0.636
Glycine	115.71	106.65	112.25	139.32	7.217	0.422	0.352	0.180
Histidine	885.04	897.45	793.71	1308.25	80.460	0.095	0.137	0.091
Phenylalanine	122.14	152.38	128.81	139.12	8.734	0.670	0.620	0.516
Proline	47.82	51.95	47.33	49.25	2.985	0.957	0.954	0.796
Serine	60.30	78.68	69.33	70.52	3.692	0.399	0.370	0.239
Tyrosine	33.01	42.46	35.67	37.90	1.819	0.321	0.448	0.257
Ornithine	152.21	124.66	266.26	169.03	22.850	0.135	0.342	0.824
Citrulline	18.42	23.89	18.86	21.54	1.486	0.567	0.630	0.541

SEM is the pooled standard error between five groups, the *P*-value indicates significance.

As shown in Table 11, dietary supplementation with RP did not significantly affect saturated fatty acid (SFA) content in the longissimus dorsi muscle of Hu sheep (*P* > 0.05). However, notable modulations were observed in specific unsaturated fatty acids (UFAs). Increasing RP inclusion elicited quadratic tendencies in C16:1, C18:1, N-3 PUFAs, C18:2N6, N-6 PUFAs, and PUFAs (*P* = 0.065, *P* = 0.087, *P* = 0.51, *P* = 0.067, *P* = 0.081, *P* = 0.079). Linear tendencies were detected for C22:5N3 and C22:6N3 (*P* = 0.053 and *P* = 0.057), while significant linear responses occurred in C24:1, N-3 PUFAs, and C20:2N6 (*P* = 0.025, *P* = 0.028, *P* = 0.017). Notably, the RP4 group exhibited significantly higher N-3 PUFA levels compared to RP0 (*P* ≤ 0.05), extremely elevated C20:2N6 levels versus the control (*P* ≤ 0.01), and superior C18:3N3 concentrations relative to RP0, RP2, and RP8 (*P* ≤ 0.05).

**TABLE 11:** Effects of dietary rose pomace supplementation on fatty acid composition in lamb longissimus dorsi muscle (μg/g) (*n* = 5).

Item	RP0	RP2	RP4	RP8	SEM	*P*-value		
						Contrast	Linear	Quadratic
C12:0	0.80	0.65	3.00	0.53	0.526	0.304	0.696	0.465
C14:0	51.60	41.14	100.33	36.72	11.516	0.183	0.840	0.435
C15:0	9.18	9.04	17.26	8.15	1.670	0.180	0.650	0.338
C16:0	665.28	607.28	828.17	530.67	47.915	0.153	0.712	0.321
C17:0	33.19	33.99	54.16	30.59	4.040	0.135	0.625	0.257
C18:0	664.39	592.13	885.93	600.82	55.062	0.201	0.816	0.569
C20:0	4.43	4.35	6.07	4.34	0.3404	0.197	0.575	0.427
C22:0	1.15	1.16	1.40	1.17	0.500	0.243	0.438	0.446
C23:0	1.62	1.66	1.75	1.63	0.219	0.132	0.397	0.150
C24:0	1.09	1.09	1.18	1.04	0.243	0.282	0.934	0.258
SFA	1432.74	1292.49	1899.25	1215.66	117.668	0.163	0.994	0.418
C16:1	35.24	31.40	56.57	24.66	5.092	0.132	0.956	0.065
C18:1	975.00	917.73	1332.44	852.52	82.974	0.168	0.884	0.087
C20:1	4.46	4.22	5.59	4.46	0.237	0.168	0.492	0.641
C24:1	1.08	1.13	1.17	1.20	0.195	0.149	0.025	0.961
MUFA	1015.77	954.50	1395.77	882.84	87.982	0.163	0.891	0.350
C18:3N3/ALA	7.95^b^	9.13^b^	14.04^a^	8.76^b^	0.835	0.026	0.181	0.119
C20:3N3	1.97	2.10	2.05	2.02	0.028	0.420	0.479	0.167
C20:5N3/EPA	2.45	2.72	2.66	2.76	0.107	0.773	0.360	0.774
C22:5N3/DPA	10.16	12.16	11.33	11.75	0.291	0.069	0.053	0.149
C22:6N3/DHA	2.62	2.74	2.90	3.06	0.079	0.255	0.057	0.614
N-3 PUFA	25.15^b^	28.86^ab^	32.97^a^	28.35^ab^	0.929	0.015	0.028	0.051
C18:2N6	373.79	435.23	524.53	401.06	21.433	0.055	0.204	0.067
C18:3N6	4.42	4.20	4.92	4.17	0.160	0.336	0.999	0.614
C20:2N6	4.40^cB^	5.4^abAB^	6.11^aA^	5.09^bcAB^	0.192	0.005	0.017	0.012
C20:3N6/DGLA	10.64	12.29	11.38	11.52	0.294	0.281	0.335	0.178
C20:4N6/AA	151.15	166.98	154.84	160.81	5.444	0.779	0.650	0.612
C22:4N6	17.27	19.21	18.38	18.76	0.711	0.825	0.519	0.619
C22:5N6	9.29	9.13	8.86	10.03	0.333	0.676	0.627	0.358
N-6 PUFA	570.97	652.44	729.03	611.44	23.963	0.103	0.205	0.081
N-6/N-3	22.42	22.61	22.15	21.51	0.391	0.799	0.464	0.516
PUFA	596.12	681.29	761.99	639.79	24.807	0.096	0.192	0.079

^A,B^Different superscripts indicate significant differences within a row (*P* < 0.01). ^a,b,c^Different superscripts indicate significant differences within a row (*P* < 0.05). SEM is the pooled standard error between five groups; the *P*-value indicates significance. SFAs, saturated fatty acids; MUFAs, monounsaturated fatty acids; N-3 PUFAs, N-3 (omega-3) polyunsaturated fatty acids; N-6 PUFAs, N-6 (omega-6) polyunsaturated fatty acids; N-6/N-3, N-6 to N-3 polyunsaturated fatty acid ratio; PUFAs, polyunsaturated fatty acids.

### 3.5 Correlation analyses

**FIGURE 5:**
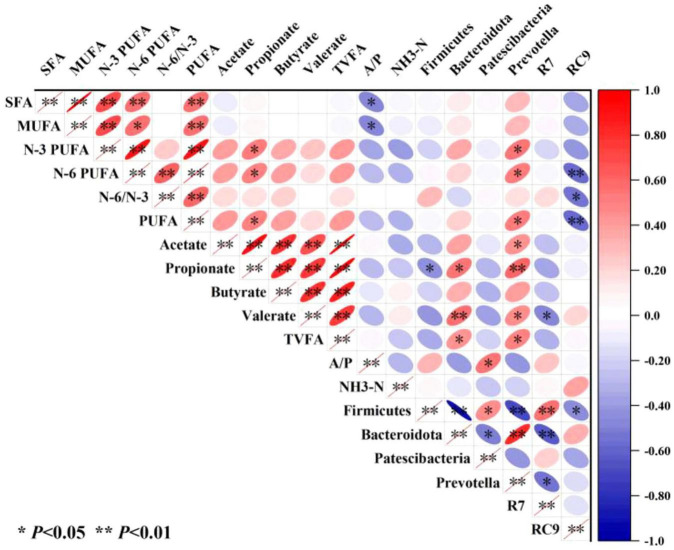
Heatmap analysis of correlations among longissimus dorsi fatty acids, rumen microbiota, and fermentation parameters. R7, the *Christensenellaceae_R-7_group*; RC9, the *Rikenellaceae_RC9_gut_group*; and A/P the acetate/propionate ratio.

The correlation analysis heatmap constructed using Pearson’s algorithm (Figure 5) revealed complex interrelationships among fatty acids in the longissimus dorsi muscle, rumen microbiota, and rumen fermentation parameters. SFAs and MUFAs exhibited significant negative correlations (*P* ≤ 0.05) with the A/P, whereas N-3 PUFAs, N-6 PUFAs, and PUFAs showed significant positive correlations (*P* ≤ 0.05) with increased propionate concentration and relative abundance of *Prevotella*. Notably, N-6 PUFAs and PUFAs were strongly negatively correlated (*P* ≤ 0.01) with the *Rikenellaceae_RC9_gut_group*, while the N-6/N-3 ratio displayed a significant negative association (*P* ≤ 0.05) with the Rikenellaceae_RC9_gut_group. Acetate positively correlated with *Prevotella* (*P* ≤ 0.05), whereas propionate exhibited divergent associations—negatively with Firmicutes (*P* ≤ 0.05) and positively with Bacteroidota (*P* ≤ 0.05) and *Prevotella* (*P* ≤ 0.01). Valerate demonstrated a highly significant positive correlation with Bacteroidota (*P* ≤ 0.01) and *Prevotella* (*P* ≤ 0.05), but a negative correlation with the *Rikenellaceae_RC9_gut_group*. TVFAs were positively linked to both Bacteroidota and *Prevotella* (*P* ≤ 0.05), and the A/P ratio was significantly associated with increased Patescibacteria abundance. These findings collectively suggest that RP-induced shifts in rumen microbiota (e.g., *Prevotella*-Bacteroidota synergism and *Rikenellaceae_RC9_gut_group* suppression) mediate lipid metabolism remodeling in muscle tissue.

## 4 Discussion

The bioactive components in rose pomace, particularly its total flavonoids, have demonstrated significant biological activities *in vitro* (Butkevièiûtë et al., 2022). Known for their potent antioxidant properties, flavonoids can mitigate cellular oxidative damage by scavenging free radicals (Olagaray and Bradford, 2019), potentially enhancing growth efficiency and health status in lambs. However, the present study revealed that dietary inclusion of 2–8% rose pomace did not significantly improve growth performance or slaughter parameters in Hu sheep, despite the increased DMI observed in the RP8 group. This discrepancy may stem from the dual regulatory effects of RP as a plant-derived byproduct-its high fiber content and phytochemical complexity likely exert counterbalancing influences on ruminant digestion (Mallick et al., 2024). The increase in DMI in the RP8 group might be related to the fact that the fibrous components promoted rumen filling, improved palatability, and the rose petal residue had a pleasant aroma, which encouraged the test sheep to eat more. However, due to its relatively low nutrient content, it did not translate into weight gain.

Rumen fermentation constitutes the core metabolic process in ruminant digestion, with volatile fatty acids (VFAs) serving as the primary energy source and directly promoting efficient host energy metabolism (Li Q. et al., 2025). Acetate, a major VFA derived from carbohydrate fermentation (particularly cellulose and hemicellulose degradation) (Liang et al., 2021), exhibited a linear increase with rising RP inclusion in this study. The high fiber content of RP may stimulate fibrolytic bacterial activity at moderate doses, while excessive levels could inhibit fermentation due to antinutritional factor accumulation (Wora-anu et al., 2007). Propionate, another critical VFA involved in gluconeogenesis and energy provision (Salisbury et al., 2021), also increased linearly with RP addition. This rise may correlate with flavonoids and terpenoids in RP modulating the rumen microenvironment to favor propionate-producing microbiota (Yu et al., 2023). Concurrent linear increases in isobutyrate, butyrate, isovalerate, and valerate collectively contributed to elevated total VFA levels. RP supplementation reduced ruminal ammonia nitrogen NH_3_-N concentration, though this effect diminished in the RP8 group. This dose-dependent phenomenon may reflect polyphenol-mediated mechanisms: at low-to-moderate doses, polyphenols likely suppress proteolytic bacteria or bind ammonia precursors to reduce protein degradation (Frazier et al., 2010); at high doses, microbial adaptation or fiber substrate buffering may counteract inhibitory effects (Mithul Aravind et al., 2021).

The rumen microbiota play a critical role in nutrient digestion, rumen fermentation, and host health maintenance, fulfilling up to 90% of the metabolic demands in ruminants (Liu et al., 2023). In this study, dietary supplementation with RP did not significantly affect alpha-diversity indices of the rumen microbiota in Hu sheep, indicating that addition of increasing amounts of RP did not alter the microbial diversity or richness. These results align with OTU compositional analysis, which further supports the conclusion that RP supplementation minimally affected the core structure of the rumen microbial community. The PCoA plot revealed close clustering of samples, suggesting little change in rumen bacterial compositions across groups. This phenomenon may stem from the complex interdependencies among rumen microorganisms, wherein RP’s bioactive components potentially maintain rumen homeostasis and microbial evenness.

The structural dynamics of rumen microbiota are a critical factor influencing nutrient metabolism and host health in ruminants. This study revealed that Firmicutes and Bacteroidota were the dominant phyla in the rumen of Hu sheep, consistent with previous findings in ruminants by Zhang et al. (2018). At the phylum level, the relative abundance of Firmicutes decreased linearly with increasing RP supplementation, while Bacteroidota showed a significant linear increase. This reciprocal relationship may reflect metabolic complementarity: the decline in Firmicutes—key cellulolytic bacteria—could be associated with physical barriers formed by lignin-cellulose complexes in RP- or flavonoid-mediated interference with bacterial cell wall synthesis (Cui et al., 2023). Conversely, the increase in Bacteroidota—a major group for complex carbohydrate degradation—may result from enhanced enzymatic activities driven by RP’s high cellulose and pectin content (Grondin et al., 2017). At the genus level, dominant taxa included the *Prevotella*, *Christensenellaceae_R-7_group*, the *Rikenellaceae_RC9_gut_group*, and the *Lachnospiraceae_NK3A20_group*. The quadratic response of the *Rikenellaceae_RC9_gut_group* abundance to RP supplementation may be linked to its modulation of microbial competition dynamics. At lower supplementation levels, these genera might be competitively inhibited by other bacteria, whereas increased RP inclusion alters the rumen microenvironment, enabling the *Rikenellaceae_RC9_gut_group* to better compete for resources, thereby increasing its relative abundance.

The protein content and amino acid composition in ruminants is influenced by dietary factors and closely associated with microbial metabolism (An et al., 2024). In this study, the predominant amino acids in longissimus dorsi muscle were glutamine (Gln), histidine (His), alanine (Ala), threonine (Thr), and arginine (Arg). Under our experimental conditions, dietary supplementation with increasing levels of RP did not significantly alter amino acid profiles. Histidine exhibited a quadratic trend, but failed to reach statistical significance. The absence of definitive correlations between RP inclusion and amino acid content may result from multiple factors, including limited supplementation doses, inherent physiological regulation in lambs, and the unique physicochemical properties of RP components.

A total of 26 fatty acids were detected in the longissimus dorsi muscle of Hu sheep, comprising 10 SFAs, 4 MUFAs, and 12 PUFAs. Across all groups, the hierarchical composition followed ΣSFA > ΣMUFA > ΣPUFA, consistent with the general fatty acid profile reported by Roberta et al. (de Valença et al., 2024) and Jia et al. (Jia et al., 2024). SFAs primarily contribute to energy storage and cellular membrane structure, whereas PUFAs are critically involved in membrane fluidity, immune regulation, and inflammatory response modulation (Mititelu et al., 2025). The RP4 group exhibited significantly higher levels of C20:2N6 and N-3 PUFAs compared to the RP0, indicating that moderate RP supplementation enhanced the deposition of these fatty acids. N-3 PUFAs, long recognized for their physiological benefits, improve membrane stability, moderate immune responses, attenuate excessive inflammation, and reduce the risk of systemic inflammatory syndrome (Zinkow et al., 2024). Although, excess N-6 PUFA accumulation may disrupt cholesterol metabolism and elevate cardiovascular disease susceptibility (Yang et al., 2023), increasing N-3 PUFA content in muscle holds significant promise for improving the nutritional value of lamb as an addition to a healthful diet. Vlaicu et al. (2022) demonstrated that rose hip supplementation of feed for laying hens elevated n-3 PUFA content in eggs and improved storability, likely mediated by polyphenol-induced inhibition of lipid peroxidation and PUFA degradation. Our findings align with these observations, suggesting that antioxidant polyphenols in rose pomace may similarly protect PUFAs from oxidative loss in sheep muscle. Notably, the RP4 group showed marked increases in the C20:2N6, N-3 PUFAs, and C18:3N3/ALA content, whereas the RP8 group failed to exhibit dose-dependent improvements. This implies an optimal RP inclusion level (4% in this study), above which the higher dose will not further enhance fatty acid profiles and could even negatively affect rumen microbiota and nutrient utilization. The 4% RP addition likely balances the polyphenol-mediated antioxidant effects with potential inhibitory actions on rumen fermentation, thereby serving as a strategic intervention to optimize Hu sheep meat quality.

The nutritional metabolism of ruminants is closely linked to their unique rumen fermentation system, where microbial communities regulate host nutrient utilization efficiency through multi-layered metabolic networks (Zhao et al., 2024). Correlation analysis integrating rumen metabolites, microbiota, and longissimus dorsi fatty acids (Figure 5) revealed that N-3 PUFAs in muscle correlated with ruminal propionate content and Prevotella abundance. Propionate levels were influenced by the relative abundances of Firmicutes, Bacteroidota, and *Prevotella*, indicating that RP supplementation could enhance N-3 PUFA deposition in muscle by increasing Bacteroidota abundance and thereby elevating propionate production. The Bacteroidota (particularly *Prevotella*) possesses efficient fiber-degrading enzymatic systems, and their increased abundance may promote the breakdown of complex polysaccharides in RP, generating pyruvate metabolic intermediates that enhance propionate synthesis (Salyers et al., 1978). Propionate may regulate lipid metabolism through the GPR41/43 receptor-mediated MAPK signaling pathway or by inhibiting histone deacetylase (HDAC) activity to alter gene expression (Deja et al., 2024) (Li et al., 2018), although the specific details of these mechanisms require further investigation. Additionally, the N-6 PUFA levels were associated not only with propionate concentration and *Prevotella* abundance, but also negatively correlated with *Rikenellaceae_RC9_gut_group* abundance. Despite significant increases in propionate, N-6 PUFA content did not reach statistical significance, possibly because of reduced abundance of *the Rikenellaceae_RC9_gut_group*.

## 5 Conclusion

Under our experimental conditions, dietary supplementation with 2–4% rose pomace generated optimal rumen fermentation and improved the quality of meat from Hu lambs, effectively enhancing ruminal acetate, propionate, isobutyrate, valerate, and total volatile fatty acid (TVFA) levels, while increasing functional fatty acids (C18:3N3/ALAs, N-3 PUFAs, and C20:2N6) in the longissimus dorsi muscle. We conclude that rose pomace, the waste byproduct of rose oil production, can be utilized as a beneficial plant-derived feed additive, with a recommended dietary inclusion level of 2–4%.

## Data Availability

The rumen bacterial data were deposited into the National Center for Biotechnology Information (NCBI) Sequence Read Archive (SRA) with the accession numbers PRJNA1230601. The other original data were uploaded to Figshare: DOI: 10.6084/m9.figshare.29184182.

## Footnotes

1
www.biocloud.net
